# Exploring the Limits of the Geometric Copolymerization Model

**DOI:** 10.3390/polym9030101

**Published:** 2017-03-13

**Authors:** Martin S. Engler, Kerstin Scheubert, Ulrich S. Schubert, Sebastian Böcker

**Affiliations:** 1 Life Sciences Group, Centrum Wiskunde & Informatica, Science Park 123, 1089XG Amsterdam, The Netherlands; martin.engler@cwi.nl; 2 Chair of Bioinformatics, Friedrich Schiller University, Ernst-Abbe-Platz 2, 07743 Jena, Germany; kerstin.scheubert@uni-jena.de; 3 Laboratory of Organic and Macromolecular Chemistry (IOMC), Friedrich Schiller University Jena, Humboldtstr. 10, 07743 Jena, Germany; ulrich.schubert@uni-jena.de; 4 Jena Center for Soft Matter (JCSM), Friedrich Schiller University Jena, Philosophenweg 7, 07743 Jena, Germany

**Keywords:** copolymer kinetics, copolymer fingerprint, Markov model, Monte Carlo simulations

## Abstract

The geometric copolymerization model is a recently introduced statistical Markov chain model. Here, we investigate its practicality. First, several approaches to identify the optimal model parameters from observed copolymer fingerprints are evaluated using Monte Carlo simulated data. Directly optimizing the parameters is robust against noise but has impractically long running times. A compromise between robustness and running time is found by exploiting the relationship between monomer concentrations calculated by ordinary differential equations and the geometric model. Second, we investigate the applicability of the model to copolymerizations beyond living polymerization and show that the model is useful for copolymerizations involving termination and depropagation reactions.

## 1. Introduction

Theoretical models for copolymerization of linear binary copolymers of two monomer types A and B are well established in polymer science. Very recently, we introduced a new statistical model [1]. Here, we investigate its limits with regard to different polymerization types and evaluate several methods to determine the model parameters.

Mass spectrometry (MS) is frequently applied to characterize (*co*-)polymers, in particular matrix-assisted laser desorption/ionization time-of-flight MS [2,3]. Mass spectra can be transformed to copolymer fingerprints [4,5,6,7], which represent the two-dimensional distribution of all copolymer chains. A copolymer fingerprint shows the abundance of each possible combination of monomer numbers. This work focuses on copolymer fingerprints of linear binary copolymers.

Several theoretical models for copolymerization were devised in the past, starting with Mayo and Lewis and their terminal model [8], which describes four propagation reactions and is determined by the monomer reactivity ratios. Computational approaches to such a model can be categorized into three types: ordinary differential equations (ODEs), Markov chains, and Monte Carlo methods.

By applying ODEs to the terminal model, Mayo and Lewis deduced the copolymer equation [8], which provides the copolymer composition. Using population balance equations and ODE systems, Kryven and Iedema were able to extract simple sequence patterns, but not the full distribution of sequences [9]. Markov and Hidden Markov models are frequently applied in the field of synthetic polymers and biopolymers [10,11,12]. The terminal model can be represented as a Markov chain in a straightforward way [13], which enables the probability of a single copolymer chain, but not the distribution of all chains, to be computed. Monte Carlo methods can be used to simulate chemical reactions [14]. In polymer science, Monte Carlo simulations have been evaluated against experimental data [15,16,17] and used to compute copolymer fingerprints [18,19,20,21]. However, Monte Carlo simulations can be time- and memory-intensive.

In a recent publication, we proposed several variants of a new Markov chain model to characterize the whole distribution of copolymer chains based on a simple living copolymerization scheme [1]. Unlike the traditional terminal model [8], the model allows for variable chain lengths and time-dependent monomer probabilities to model copolymer chain length distributions and differential monomer conversion rates, respectively. In contrast to Monte Carlo simulations, the model is exact and deterministic. In particular, it allows for calculating the exact likelihood of any polymer chain. Monte Carlo simulations provide a random sample, which converges to the true copolymer distribution with an increasing number of simulated chains. However, running times and memory requirements correlate with the number of simulated chains. We showed that the proposed model is faster and requires less memory [1].

The model is a Markov chain with discrete time steps, so-called synthesis steps. Let us consider a single polymer chain. In each synthesis step, the chain is propagated by some number k≥0 of monomers. To this end, the probabilities of all possible propagation events are calculated from three different probabilities.

First, the probability of adding *k* monomers, which is constant for each synthesis step, has to be calculated using either a Bernoulli or geometric probability distribution. This leads to a binomial or negative binomial distribution of polymer lengths. The negative binomial is the discrete equivalent to the gamma (Schulz–Zimm) distribution. This is in agreement with the literature, where several distributions for modeling chain lengths can be found: most probable (Schulz–Flory), gamma, Poisson, or hypergeometric distributions [13,22,23]. All these distributions are related. On the one hand, for large chain lengths, the most probable distribution approximates the gamma distribution, while the gamma and binomial distributions approximate the Poisson distribution for large chain lengths. On the other hand, the gamma and Poisson distributions are the limiting cases of the hypergeometric chain length distribution [23].

Second, the probability of the polymer chain colliding with *x* A-monomers and *y* B-monomers, where x+y=k has to be calculated. This probability may change between synthesis steps to reflect differential monomer conversion rates.

Third, basic collision theory has to be considered. In order for a collision of two molecules to be successful, the collision energy needs to be higher than the activation energy. As a consequence, the geometric model needs to incorporate the probability of a successful collision between two monomers.

In our recent publication [1], Monte Carlo simulated data was used to evaluate four variants of the model: The number of added monomers following a Bernoulli or geometric distribution and either using reactivity parameters or not. This work focuses on the variant which was the most accurate, the Geometric model with reactivity parameters. In our previous paper, we suggested that it is possible to estimate the model parameters from the observed copolymer fingerprints [1]. In this paper, we show that determining the model parameters from copolymer fingerprints is a challenging optimization problem. First, several methods are presented to increase the accuracy of the results and to decrease the running times. Several general purpose optimization algorithms and the robustness of the proposed methods against measurement noise are evaluated. Second, the accuracy of the geometric model is evaluated using different copolymerization types beyond living polymerization: reversible living polymerization, controlled radical polymerization, and free radical polymerization. The evaluation uses fingerprints and copolymer chains computed by Monte Carlo simulations.

## 2. Methods

In the following, we briefly explain the Geometric copolymerization model and the experimental setup. First, copolymer fingerprints using Monte Carlo methods were computed (see below). Different noise levels were simulated by multiplying the fingerprint abundances by log-normal distributed random noise with mean zero and variance *σ*, where the noise parameter *σ* has the values 0, 0.05, 0.15, and 0.25. Then, the model parameter optimizations were performed in parallel on a compute cluster of four 2.4 GHz CPUs with 16 cores each and 6 GB RAM per process. The optimization algorithms are single-threaded, thus the reported running times are similar to the running times to be expected when performing an analysis on a standard laptop. Finally, the log likelihoood ratios were computed as described below.

### 2.1. The Geometric Copolymerization Model

In the following, let the matrix *M* of size n×m be a *copolymer fingerprint*, in which entry $M_{a,b}$ gives the relative abundance of a copolymer with *a* monomers of type A and *b* monomers of type B. The geometric copolymerization model is Markov chain with discrete time steps, so-called synthesis steps. The states of the Markov chain correspond to the fingerprint entries $M_{a,b}$. However, to incorporate the reactivity parameters, states $M_{a,b}$ have to be divided into $M_{a,b}^{A}$, copolymer chains with *a* A-monomers and *b* B-monomers ending in A, and $M_{a,b}^{B}$, copolymer chains with *a* A-monomers and *b* B-monomers ending in B, with $M_{a,b}=M_{a,b}^{A}+M_{a,b}^{B}$. In addition, the initiator state *I* is defined as $I=M_{0,0}$.

**Figure 1:**
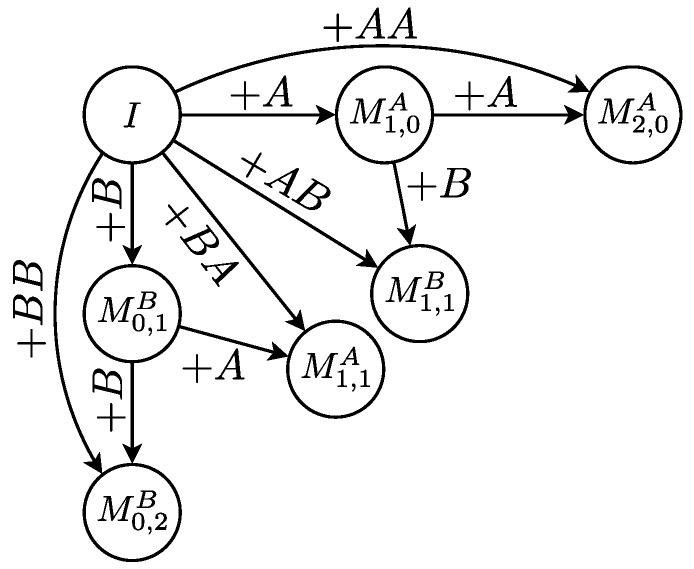
All possible transitions for copolymer chain lengths ≤2. For example, the transition from the initiator state *I* to the state $M_{2,0}^{A}$ (copolymer chains having two A-monomers and ending in A) corresponds to adding the sequence AA. Note that transitions that add more than two monomers correspond to multiple events. For example, the transition of *I* to $M_{2,1}^{A}$ corresponds to adding the two sequences BAA and ABA.

In each synthesis step, the probabilities for all possible transitions $M_{a,b}^{X}$ to $M_{a+x,b+y}^{Y}$ and *I* to $M_{x,y}^{Y}$ are calculated for X,Y∈{A,B} and all *a*, *b*, *x* and *y* (Figure 1). These probabilities are determined by the probability of adding k=x+y monomers, the probabilities of adding *x* A-monomers and *y* B-monomers and the reactivity ratios. To compute a fingerprint using the model, the states of the Markov chain are initialized as I=1 and $M_{a,b}^{X}=0$ for X∈{A,B} and all *a* and *b*. Subsequently, all possible transitions are applied in each synthesis step 1≤t≤T. Finally, the fingerprint $M=M^{A}\left(T\right)+M^{B}\left(T\right)$ is computed.

### 2.2. Monte Carlo Reaction Schemes

We evaluate our methods against Monte Carlo simulations of different polymerization types (Table 1): living polymerization (LP), reversible living polymerization (RLP), free radical polymerization (FRP), and controlled radical polymerization (CRP).

For living polymerization, the following reaction scheme were used. An active center is donated as X^•^, and a polymer chain ending with X as ∼ X, where X can be one of the monomers A or B, or initiator I. Two types of reactions, initiation and propagation reactions were modeled:

$$\begin{array}{c} I+A\overset{k_{IA}}{\to }\;∼A^{•}\\ I+B\overset{k_{IB}}{\to }\;∼B^{•} \end{array}\}\mathrm{Initiation}$$

$$\begin{array}{c} ∼A^{•}+A\overset{k_{AA}}{\to }\;∼A^{•}\\ ∼A^{•}+B\overset{k_{AB}}{\to }\;∼B^{•}\\ ∼B^{•}+A\overset{k_{BA}}{\to }\;∼A^{•}\\ ∼B^{•}+B\overset{k_{BB}}{\to }\;∼B^{•} \end{array}\}\mathrm{Propagation}.$$

For reversible living polymerization, the initiation and propagation reactions of the living polymerization and additionally the following depropagation reactions were used:

$$\begin{array}{c} ∼{\mathrm{IA}}^{•}\overset{k_{IA}^{d}}{\to }\;∼I^{•}+A\\ ∼{\mathrm{IB}}^{•}\overset{k_{IB}^{d}}{\to }\;∼I^{•}+B\\ ∼{\mathrm{AA}}^{•}\overset{k_{AA}^{d}}{\to }\;∼A^{•}+A\\ ∼{\mathrm{AB}}^{•}\overset{k_{AB}^{d}}{\to }\;∼A^{•}+B\\ ∼{\mathrm{BA}}^{•}\overset{k_{BA}^{d}}{\to }\;∼B^{•}+A\\ ∼{\mathrm{BB}}^{•}\overset{k_{BB}^{d}}{\to }\;∼B^{•}+B \end{array}\}\mathrm{Depropagation}.$$

For free and controlled radical polymerization, the initiation and propagation reactions of the living polymerization and, additionally, model chain termination by recombination and disproportionation were used:

$$\begin{array}{c} ∼A^{•}+∼A^{•}\overset{k_{AA}^{r}}{\to }\;∼\mathrm{AA}∼\\ ∼A^{•}+∼B^{•}\overset{k_{AB}^{r}}{\to }\;∼\mathrm{AB}∼\\ ∼B^{•}+∼B^{•}\overset{k_{BB}^{r}}{\to }\;∼\mathrm{BB}∼ \end{array}\}\mathrm{Recombination}$$

$$\begin{array}{c} ∼A^{•}+∼A^{•}\overset{k_{AA}^{dp}}{\to }\;∼A+∼A\\ ∼A^{•}+∼B^{•}\overset{k_{AB}^{dp}}{\to }\;∼A+∼B\\ ∼B^{•}+∼B^{•}\overset{k_{BB}^{dp}}{\to }\;∼B+∼B \end{array}\}\mathrm{Disproportionation}.$$

For free radical polymerization, the initiation and propagation reactions of the living polymerization, chain termination by recombination and disproportionation, and the following additional initiation reaction to model a decomposing initiator complex were used:

$$I_{2}\overset{k^{dec}}{\to }2\cdot I\}\mathrm{Initiator}\;\mathrm{Decomposition}.$$

For controlled radical polymerization, the initiation and propagation reactions of the living polymerization, chain termination by recombination and disproportionation, and the following additional activation and deactivation reactions were used:

$$\begin{array}{c} \mathrm{IX}+L\overset{k_{I}^{a}}{\to }I+\mathrm{LX}\\ ∼\mathrm{AX}+L\overset{k_{A}^{a}}{\to }\;∼A^{•}+\mathrm{LX}\\ ∼\mathrm{BX}+L\overset{k_{B}^{a}}{\to }\;∼B^{•}+\mathrm{LX} \end{array}\}\mathrm{Activation}$$

$$\begin{array}{c} I+\mathrm{LX}\overset{k_{I}^{da}}{\to }\mathrm{IX}+L\\ ∼A^{•}+\mathrm{LX}\overset{k_{A}^{da}}{\to }\;∼\mathrm{AX}+L\\ ∼B^{•}+\mathrm{LX}\overset{k_{B}^{da}}{\to }\;∼\mathrm{BX}+L \end{array}\}\mathrm{Deactivation}.$$

### 2.3. Datasets and Monte Carlo Parameters

For all datasets, the reaction rates (Supplementary Table S1) were chosen such that $r_{A}=\frac{1}{r_{B}}=r$, with the reactivity ratios $r_{A}=\frac{k_{AA}}{k_{AB}}$, $r_{B}=\frac{k_{BB}}{k_{BA}}$, and the ratio of homopropagation rates $r=\frac{k_{AA}}{k_{BB}}$.

For living polymerization, we use three Monte Carlo simulated datasets reported in our previous paper [1]: $r_{A}=0.01$, $r_{A}=1.0$, and $r_{A}=2.0$. Two additional datasets were simulated with the same reactivity ratios $r_{A}=2.0$, but different degrees of polymerization ${DP}_{n}=25$ and ${DP}_{n}=45$, respectively. For an overview of the initial concentrations and reaction rates, please see Supplementary Table S1.

For Monte Carlo simulations of the other polymerization types, the parameters of the dataset with ${DP}_{n}=25,r_{A}=2.0$ for initiation and propagation reactions were used. The reaction rates of the termination and depropagation rates $k^{d}$, $k^{r}$, $k^{dp}$ varied over 0, 0.001, 0.01, and 0.1. For free radical polymerization, a decomposition rate $k^{DEC}=10$ were used, and for controlled radical polymerization, activation rates $k^{a}=100$ and deactivation rates $k^{da}=0.01$ were used.

### 2.4. Log Likelihood Ratio

Our model allows for computing the likelihood of a single polymer chain [24]. Let *S* be a sequence (polymer chain) and *H* a hypothesis, i.e., the geometric model. Let P(S|H) be the likelihood of *S*, given model *H*. Then, the log likelihood of a dataset *D* is:

(1) $$\log P\left(D|H\right)=\sum_{S\in D}\log P\left(S|H\right).$$

To evaluate the models, we compare the log likelihood of the data under the model to the log likelihood under the null hypothesis $H_{0}$. In the null model, all positions in the polymer chain are independent random variables. For each position *i* over all chains in the dataset, we determine the frequencies $f_{A}$ and $f_{B}$ of A and B, respectively. Let $P(s_{i})$ be the likelihood of monomer *i* in chain *S*. Then, the log likelihood of a dataset, assuming the null model, is:

(2) $$\log P\left(D|H_{0}\right)=\sum_{S\in D}\log P\left(S|H_{0}\right)=\sum_{S\in D}\sum_{i=1}^{\left|S\right|}\log P\left(s_{i}\right)=\sum_{S\in D}\sum_{i=1}^{\left|S\right|}\log\left\{\begin{array}{c} f_{A},\mathrm{if}s_{i}=A,\\ f_{B},\mathrm{if}s_{i}=B. \end{array}\right.$$

We compute the log likelihood ratio:

(3) $$\log\frac{P(D|H)}{P(D|H_{0})}=\log P\left(D|H\right)-\log P\left(D|H_{0}\right).$$

The log likelihood ratio is a “sanity check” for statistical models. If the ratio is below zero, the hypothesis should be dismissed and accepted if the ratio is above zero.

## 3. Results and Discussion

In the following, let the matrix *M* of size n×m be a *copolymer fingerprint*, in which entry $M_{a,b}$ gives the relative abundance of a copolymer with *a* monomers of type A and *b* monomers of type B. Let $f\left(p_{A},p_{AA},p_{AB},p_{BA},p_{BB}\right)=M^{c}$ be the *fingerprint-generating function*, which uses the geometric model with reactivity parameters to compute a fingerprint $M^{c}$ [1]. The model parameters are the monomer probability $p_{M}$, the reactivity probabilities $p_{AA}$, $p_{AB}$, $p_{BA}$, $p_{BB}$, and probability vector $p_{A}$ of size *T*, which describes the probability of encountering an A-monomer for each synthesis step 1≤t≤T. The probability of encountering a B-monomer is implicitly given because $p_{A}\left(t\right)+p_{B}\left(t\right)=1$. The monomer probability $p_{M}$ and the number of synthesis steps *T* can be easily computed from the copolymer length distribution [1].

Formally, the problem to solve is finding the parameters $p_{AA}$, $p_{AB}$, $p_{BA}$, $p_{BB}$, and the vector $p_{A}$, which minimize the distance of the computed fingerprint $M^{c}$ to an observed fingerprint $M^{o}$. This corresponds to optimizing the following *objective function*:

(4) $$\underset{p_{A},p_{AA},p_{AB},p_{BA},p_{BB}}{arg min}||f\left(p_{A},p_{AA},p_{AB},p_{BA},p_{BB}\right)-M^{o}{\left|\right|}_{2}.$$

The objective function computes the difference between the computed and observed fingerprints according to Equation (4). We use general purpose optimizers to identify the best parameters. The optimizers use different strategies and the running times vary greatly, in the small examples given in this work between 0.5 and 19 h (see Supplementary Figure S1). Generally, the optimization is challenging and its computation is time-demanding. First, the question needs to be answered: what are the main reasons for the long running time?

In our previous work [1], we introduced four variants of a discrete Markov chain copolymerization model. The models use either reactivity probabilities or not, and the number of added monomers per synthesis step either follows a Bernoulli or geometric distribution. A model is defined to be *order-independent* if the resulting fingerprints are the same for any permutation of its parameter $p_{A}$. The models are order-independent if the reactivity ratios are one (see the Supplementary section). Since there are T! possible

permutations, this results in T! global optima. However, for reactivity ratios of one, the ratios of monomers never change. As a consequence, $p_{A}$ is constant and there is exactly one global optimum. However, for reactivity ratios near one, the objective values of all permutations are very similar. This is challenging for the optimization algorithms and certainly contributes to the long running time of the optimization.

Another contributing factor is the size *T* of the vector $p_{A}$, resulting in a *T*-dimensional search space. *T* can be computed from the observed copolymer length distribution. The length distribution of the geometric model is a negative binomial distribution with the parameters *T* and $p_{M}$ [1]. In each of the *T* steps, the number of added monomers is geometrically distributed. Considering usual copolymer lengths, *T* can be expected to be between 10 and 100. Optimizing ∼100 variables simultaneously with a general purpose optimizer is a challenging task and certainly contributes to the long running time of the optimization.

### 3.1. Parameter Space Reduction

The two main challenges for the optimization algorithms are the very similar objective values for reactivity ratios near one and—more importantly—the large search space defined by the length of the model parameter vector $p_{A}$. We focus on the second challenge and propose two approaches to change the fingerprint-generating function in order to speed up the optimization.

The first approach is to optimize only a fraction of the *T* values in $p_{A}$ (25% in this work), and linearly interpolate all other values in between. Furthermore, we restrict the search space by forcing $p_{A}$ to be either increasing or decreasing. To this end, a decreasing $p_{A}$ is defined as:

(5) $$\begin{array}{cc} p_{A}\left(t\right) & =p\left(t\right)\cdot p_{A}\left(t-1\right),\\ p_{A}\left(1\right) & =p(1), \end{array}$$

and an increasing $p_{A}$ as:

(6) $$\begin{array}{cc} p_{A}\left(t\right) & =p_{A}\left(t-1\right)+p\left(t\right)\cdot \left(1-p_{A}\left(t-1\right)\right),\\ p_{A}\left(1\right) & =p(1). \end{array}$$

The second approach is to exploit the relationship between $p_{A}$ and monomer concentrations. We define *T* time intervals, such that the change in concentration is the same for each interval. Subsequently, the mean concentrations $\tilde{\left[A\right]}\left(t\right)$ and $\tilde{\left[B\right]}\left(t\right)$ are calculated for each interval 1≤t≤T. Then, the probability vector $p_{A}\left(t\right)$ can be calculated as:

(7) $$p_{A}\left(t\right)=\frac{\tilde{\left[A\right]}\left(t\right)}{\tilde{\left[A\right]}\left(t\right)+\tilde{\left[B\right]}\left(t\right)}.$$

There is also a relationship between the reaction rates and the reactivity model parameters. For X,Y∈{A,B}, the reactivity parameters are:

(8) $$p_{XY}=\frac{k_{XY}}{k_{XA}+k_{XB}}.$$

The second approach uses both relationships: first, an ODE system using the living copolymerization reaction scheme is solved. Second, the reactivity parameters are computed from the reaction rates and $p_{A}$ from the concentration gradient. Then, the fingerprint $M^{c}$ can be computed using the geometric model. This allows us to optimize the ODE parameters (reaction rates and initial concentrations) according to Equation (4). Thus, the dimension of the search space is constant and independent of *T*.

### 3.2. Parameter Optimization

In the following, we compare three fingerprint-generating functions: directly optimizing $p_{A}$ (Direct), interpolating $p_{A}$ (Spline), and optimizing the ODE parameters (ODE), with the spline and ODE approaches as described above. All three of the functions use the geometric model with reactivity parameters to compute the copolymer fingerprint. The transformation from the model parameters to the copolymer fingerprint is highly nonlinear. To the best of our knowledge, no special purpose solvers exist for such a function. Therefore, we have to resort to general purpose optimization algorithms. We use the algorithms implemented in the Optimization Algorithm Toolkit [25,26] and Apache Math Commons 3.2 library [27]. The algorithms use different strategies to find the best parameters and do not require computing gradients. The performance of the optimizers is application-specific and depends on the selected fingerprint-generating function.

We choose several instances with low degree of polymerization $DP_{n}=3$ and three different reactivity ratios $r_{A}$, $r_{B}$ and homopropagation ratios *r*. Please note that, for all datasets, $r_{A}=\frac{1}{r_{B}}=r$. First, we choose the reactivity ratio $r_{A}=2.0$, for which the geometric model can provide a good fit [1]. Second, we choose $r_{A}=0.01$, since this results in a copolymer with binomial-like length distribution (in contrast to a more common Schulz–Zimm-like distribution), which should be more challenging for the geometric model [1]. Third, we choose $r_{A}=1.0$. This results in constant monomer concentrations and, thus, the optimal $p_{A}$ is also constant. This means that the optimum lies on the parameter space limits when using the spline fingerprint-generating function, which should be a challenging task for the optimizers. Furthermore, we also select two instances with $r_{A}=2.0$ and higher degrees of polymerization $DP_{n}=25$ and $DP_{n}=45$, which are copolymer lengths to be expected in practice.

First, we choose the top three algorithms with highest log likelihood ratio for each fingerprint-generating function (Table 2). To this end, all algorithms are evaluated on the $DP_{n}=3,r_{A}=2.0$ dataset without noise (see Supplementary Figures S1–S3) and the log likelihood ratios of the results are calculated. In addition to comparing the log likelihoods, the ratio also acts as a “sanity check” for the model parameterizations. The ratio compares the likelihoods to the likelihood of a null hypothesis. The null hypothesis assumes that all positions are independent random variables. If the log likelihood ratio is below zero, the null model has a higher likelihood and the parameterization should be dismissed.

**Figure 2:**
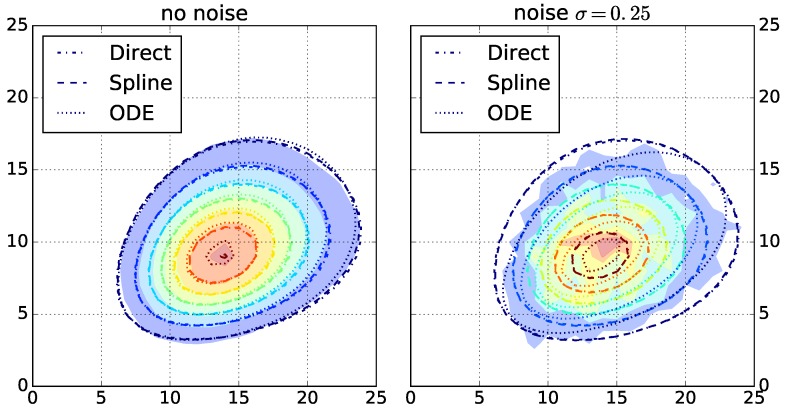
Filled contours: copolymer fingerprints of $DP_{n}=25$ computed by Monte Carlo simulations with no (**left**) and high applied noise (**right**). Contours: fingerprints computed by the geometric model using the best parameters computed by the optimization algorithms for each of the fingerprint-generating functions (direct, spline, and ODE).

After selecting the top three algorithms for each fingerprint-generating function, we evaluate the robustness of the chosen algorithms. We run the top three algorithms for each function on the other dataset with increasing simulated noise. The highest noise level with σ=0.25 results in strongly perturbed data (Figure 2 and Supplementary Figures S4–S6). For each resulting parameterization, we rank the top three algorithms by their log likelihood ratio and count the ranks for all instances (Table 2). No algorithm outperforms its rivals. Therefore, in the following, we use all chosen algorithms.

**Figure 3:**
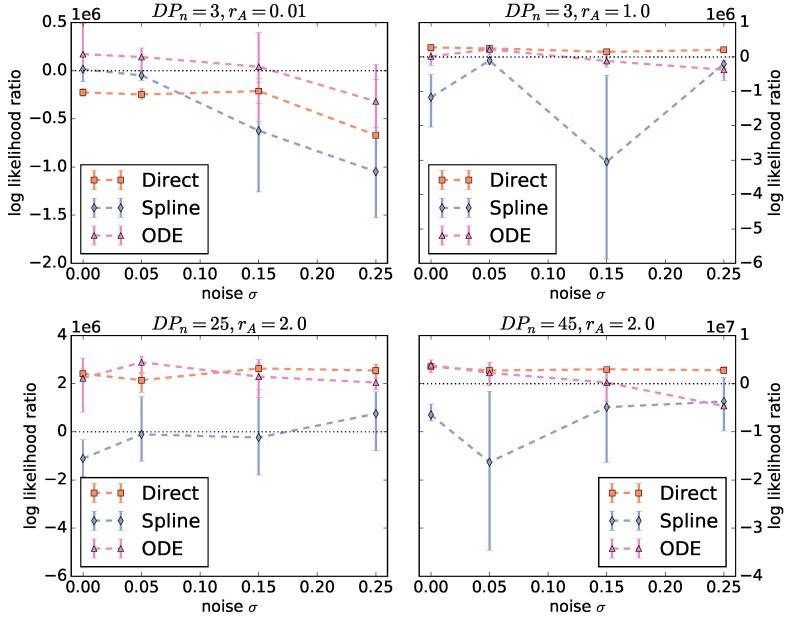
Log likelihood ratios of the results computed by the optimization algorithms as a function of noise. The ratios are averaged over all three algorithms for each fingerprint-generating function (direct, spline, ODE). The higher the ratios, the better the observed data is “explained” by the identified model parameterizations. If the ratio is below zero, the null model achieves a higher likelihood than the geometric model with the given parameterization.

To compare the three approaches (direct, spline, and ODE), we average the log likelihood ratios over all three algorithms for each fingerprint-generating function. Figure 3 shows the averaged log likelihood ratios as a function of the noise level. There are two different behaviors for $r_{A}=0.01$ and the rest of the instances. For $r_{A}=0.01$, there is a significant decrease with increasing noise and only the ODE function is able to produce a good parameterization. For the Schulz–Zimm like copolymers with $r_{A}>0.01$, the behavior of the log likelihood ratios of the ODE and direct function is not significantly different. However, for the ODE function, the range between minimum and maximum log likelihood ratio is larger and the ratio decreases more with increasing noise. Thus, using the ODE function is less robust against noise than the direct method. Unexpectedly, the optimizers using the spline function fail on all instances and result in ratios below zero in almost all cases.

**Figure 4:**
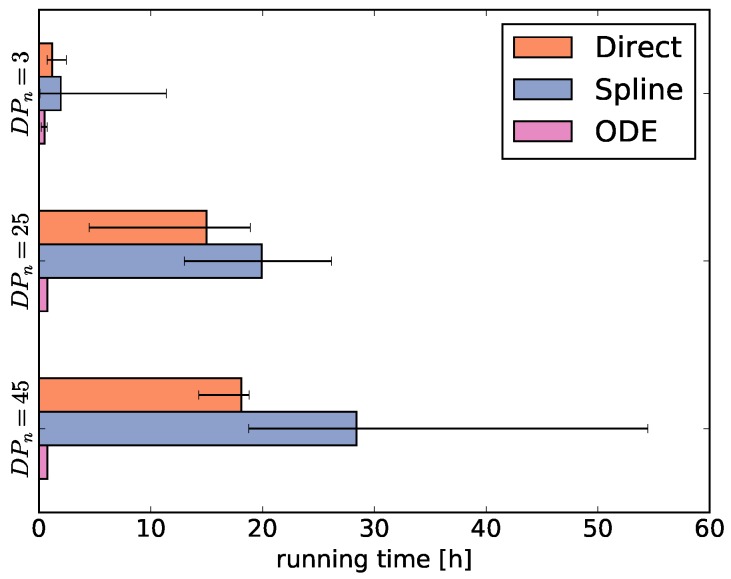
Running times of the optimizations averaged over all datasets with degree of polymerization $DP_{n}$ = 3, 25, and 45 for each fingerprint-generating function (direct, spline, ODE).

Then, we average the running times for each fingerprint-generating function for each degree of polymerization $DP_{n}$ = 3, 25, and 45 (Figure 4). As the running times largely depend on the selected optimization algorithms, the comparison of running times between the fingerprint-generating functions should be taken with a grain of salt. This means that using different optimizers may shift the numbers, but we can still infer general trends from Figure 4.

The running times of the optimizers using the direct and ODE functions behave as expected. The running time using the direct function increases with the degree of polymerization because the size of $p_{A}$ increases. Thus, the number of parameters increases, the main reason for the long running time. In contrast, the ODE function always has the same number of parameters and therefore the running time is independent of the degree of polymerization. Different from our expectations, using the spline function results in even higher running times than using the direct function, despite optimizing only a fraction of the $p_{A}$ parameter values and using the generally fast optimizers CMAES and GEO (see Supplementary Figures S1–S3).

### 3.3. Beyond Living Polymerization

**Figure 5:**
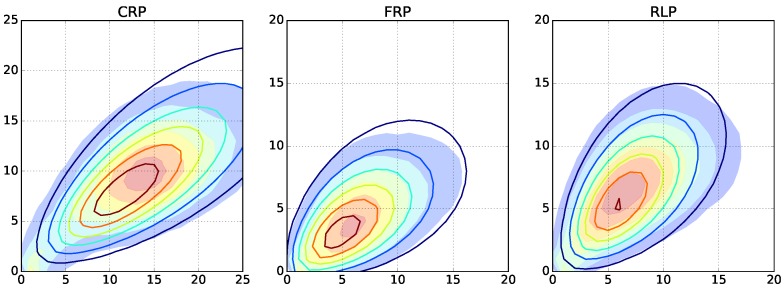
Filled contours: copolymer fingerprints of the Monte Carlo simulations of controlled radical polymerization (CRP, **left**), free radical polymerization (FRP, **center**), and reversible living polymerization (RLP, **right**) with the highest used termination and propagation reaction rates of 0.1. Contours: fingerprints computed by the model with the best parameters resulting from the optimizations using the ODE fingerprint-generating function.

Here, we investigate copolymerizations beyond a simple living polymerization. We select the $DP_{n}=25$, $r_{A}=2.0$ instance and repeatedly run Monte Carlo simulations with increasing termination and depropagation rates. For radical polymerizations, long and short length chains appear as a result of the termination by recombination and disproportionation, respectively. For free radical polymerization, the chosen decomposition rate of the initiator leads to lower average lengths. For reversible living polymerization, low length chains are appearing because of the depropagation reactions (Figure 5 and Supplementary Figures S7–S9).

**Figure 6:**
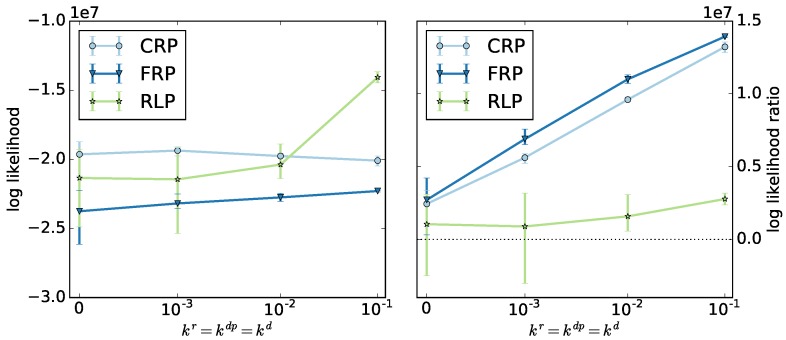
Log likelihoods (**left**) and log likelihood ratios (**right**) of the results from the optimizations using the ODE fingerprint-generating function for the controlled radical polymerization (CRP), free radical polymerization (FRP), and reversible living polymerization (RLP) as a function of termination and depropagation rates.

We select the ODE method to identify the optimal model parameters. Figure 6 shows the log likelihoods and log likelihood ratios averaged over the top three algorithms for the ODE method as a function of termination and depropagation reaction rates. The radical and reversible living polymerizations show different behaviors. For radical polymerization, the log likelihood is almost constant, but the ratio increases significantly. For reversible living polymerization, the likelihood increases significantly, but the ratio increases less.

Different from our expectations, the log likelihood ratios of all three copolymerization types increase with increasing termination and depropagation rates, due to a decreasing likelihood of the null model. We find that the geometric model can be applied for systems involving termination and depropagation reactions, even though it was designed for living copolymerization.

## 4. Conclusions

In a previous publication, we evaluated four variants of a statistical copolymerization model [1]. Here, we concentrated on the variant, which was the most accurate, the geometric model with reactivity parameters. The model computes a copolymer fingerprint.

First, the problem solve is to find the optimal model parameters from observed data. To this end, three fingerprint-generating functions were compared, which all use the model to compute the fingerprint at the end, but differ in the number of parameters. General purpose optimizers were used to find the optimal parameters for each function. Fitting the parameters using the model directly is the most robust method for copolymers with a Schulz–Zimm-like length distribution, but has a long and impractical running time. A simple approach to decrease the parameter search space using splines fails both in accuracy and in decreasing the running time. By exploiting the relationship between monomer concentration and the geometric model, we find a compromise between running time and robustness against noise. For copolymers with a binomial-like length distribution, this approach performs best. For Schulz–Zimm-like copolymers, this method is slightly less robust against noise than the direct approach, requiring good input data. However, the running time is significantly shorter. More importantly, it is independent of the degree of polymerization and, therefore, can be used for long-chained copolymers. We recommend to use this method in practice.

For those interested in the theoretical aspects, the question remains open on whether the objective function is convex and smooth. More interesting from a practical viewpoint, the geometric model allows for computing previously inaccessible statistical properties of synthesized copolymers. This will be described in a forthcoming publication. Also of interest would be extending the current model to block copolymers in a two—or more—step process, with additional intermediate fingerprints for each synthesized block.

Second, we investigated polymerizations beyond living polymerization: controlled and free radical polymerization, and reversible living polymerization. We show that the geometric model can be useful for copolymerization involving termination and depropagation reactions. Still to determine is if the model can be improved further by including termination and depropragation probabilities.

The usefulness of the model for copolymerizations beyond living polymerization is important, since these reaction systems are widely used in practice. Furthermore, termination and propagation reactions often occur accidentally in living polymerizations.

## Figures and Tables

**Table 1:** Overview of the modeled reactions types for the living polymerization (LP), reversible living polymerization (RLP), free radical polymerization (FRP), and controlled radical polymerization (CRP).

Reaction Type	LP	RLP	FRP	CRP
Initiation	×	×	×	×
Propagation	×	×	×	×
Depropagation		×		
Termination (Recomb. & Disprop.)			×	×
Initiator Decomposition			×	
(De-)Activation				×

**Table 2:** Overview of the top three optimization algorithms for each fingerprint-generating function, selected based on Supplementary Figures S1–S3. We ranked the results of the algorithms for each dataset based on the log likelihood ratios and counted the ranks.

	Algorithm	#Ranks		
		1st	2nd	3rd
Direct	Cloning, Information Gain, Aging (CLI) [28]	4	5	7
	Probabilistic Crowding (PC) [29]	6	5	5
	Restricted Tournament Selection (RTS) [30]	6	6	4
Spline	Covariance Matrix Adaptation Evolution Strategy (CMAES) [31]	3	6	7
	Deterministic Crowding (DC) [32]	3	8	5
	Generalized Extremal Optimization (GEO) [33]	10	2	4
ODE	Genetic Algorithm (GA) [34]	5	9	2
	Generalized Extremal Optimization (GEO) [33]	8	0	8
	Mutation Hill Climber (MHC) [35]	3	7	6

## Supplementary Materials

The following are available online at www.mdpi.com/2073-4360/9/3/101/s1.

## Abbreviations

The following abbreviations are used in this manuscript:

MS	Mass spectrometry
ODE	Ordinary differential equation
LP	Living polymerization
RLP	Reversible living polymerization
FRP	Free radical polymerization
CRP	Controlled radical polymerization
