# Supplementary material for: Exploring the Limits of the Geometric Copolymerization Model
Source: Polymers (Basel). 2017 Mar 13;9(3):101. doi: 10.3390/polym9030101 (PMC6431939; doi:10.3390/polym9030101)
Supplement: Supplementary file 1 [file polymers-09-00101-s001.pdf]

Supplementary Materials: Exploring the Limits of the Geometric Copolymerization Model

Martin S. Engler, Kerstin Scheubert, Ulrich S. Schubert and Sebastian Böcker

1. Monte-Carlo simulation parameters

Table S1. Initial concentrations (in mol·L^{-1}) and reaction rates of the Monte-Carlo simulations of living polymerizations.

Dataset	Initial concentration			Reaction rates			
	$[I]_0$	$[A]_0$	$[B]_0$	k_{AA}	k_{AB}	k_{BA}	k_{BB}
$DP_n = 3, r_A = 0.01$	1	1	2	0.01	1	0.01	1
$DP_n = 3, r_A = 1$	1	1	1	1	1	1	1
$DP_n = 3, r_A = 2$	1	1	2	2	1	2	1
$DP_n = 25, r_A = 2$	1	10	15	2	1	2	1
$DP_n = 45, r_A = 2$	1	20	25	2	1	2	1

2. Independence of the parameter order

In the following, let the matrix M of size $n \times m$ be a copolymer fingerprint, in which entry $M_{a,b}$ gives the relative abundance of a copolymer with a monomers of type A and b monomers of type B. Let T be the number of synthesis steps. Let p_M be the probability of encountering a monomer, and let p_A be a vector of size T with the probabilities that the encountered monomer is an A for each synthesis step $1 \leq t \leq T$. Let $p_B(t)$, the probability of encountering a monomer B be defined as $p_B(t) = 1 - p_A(t)$.

Let $\pi(x)$ be a permutation of some vector x. Let M^π be the resulting fingerprint of our model with input $\pi(p_A)$. We define a model to be *order-independent* if the resulting fingerprints are the same for any permutation of p_A, that is $M = M^\pi$ for any π.

In our previous paper, we introduced a copolymerization model with several variants similar to a discrete Markov-chain, that append monomers in each synthesis (time) step with Bernoulli or geometrically distributed probability [1]. Here, we will investigate if they are order-independent.

We do a simple experiment to investigate the order-independence of our models. For both models, we compute a fingerprint with parameters $p_A = [0, 0.1, 0.2, 0.4, 0.5]$, $p_M = 0.5$ and varying reactivity ratios. Subsequently, for all permutations $\pi(p_A)$ we compute a fingerprint and calculate the normalized root mean square error (NRMSE) in comparison to the first fingerprint (Fig. S1).

The distance between two fingerprints increases with the distance between p_A and $\pi(p_A)$. However, as the reactivity ratios approach one, the distance between the fingerprints decreases. In this experimental instance, we see that the models are order-independent if the reactivity ratios are one.

To verify that the models are order-independent for reactivity ratios of one, we investigate the model variants without reactivity parameters. As previously described by Engler *et al.* [1], for the Bernoulli model without reactivity parameters, an entry $M_{a,b}$ in the fingerprint M at synthesis step t for $a > 0$, $b > 0$, and $1 \leq t \leq T$ is given by:

$$\begin{aligned} M_{a,b}(t) = \quad & p_M \cdot p_A(t) \cdot M_{a-1,b}(t-1) \\ + \quad & p_M \cdot p_B(t) \cdot M_{a,b-1}(t-1) \\ + \quad & (1 - p_M) \cdot M_{a,b}(t-1) \end{aligned} \tag{1}$$

For the Geometric model without reactivity parameters, we first have to derive a closed form for $M_{a,b}$. Let $p_G(k)$ be the geometrically distributed probability of adding k monomers in one synthesis

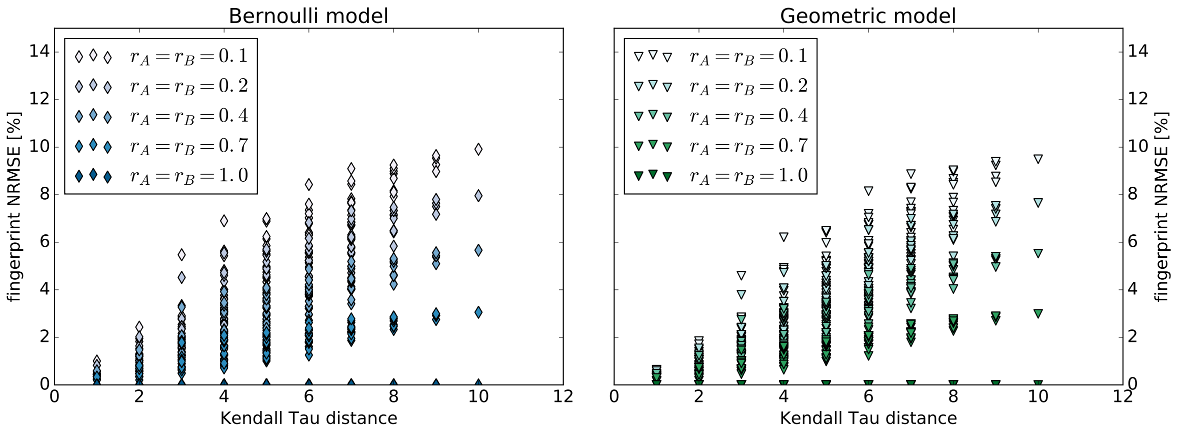

Figure S1. Normalized root mean square errors of the fingerprints for all permutations $\pi(p_{\mathsf{A}})$ compared to the fingerprint of the original p_{A} computed with the Bernoulli (left) and Geometric model (right). The Kendall Tau distance is the number of pairwise disagreements between two permutations.

step. As previously described by Engler *et al.* [1], the probability of adding i monomers A and j monomers B to a copolymer chain is given for $a > 0$, $b > 0$, and $1 \leq t \leq T$ as:

$$P(M_{a,b} \rightarrow M_{a+i,b+j}|t) = \binom{i+j}{j} \cdot P_G(i+j) \cdot p_{\mathsf{A}}(t)^i \cdot p_{\mathsf{B}}(t)^j \tag{2}$$

We define $f_{i,j}^{a,b}$ as:

$$f_{i,j}^{a,b} = \binom{a+b-i-j}{b-j} \cdot P_G(a+b-i-j) \tag{3}$$

Now we apply Eq. 2 and Eq. 3 to find a closed form expression for a fingerprint entry $M_{a,b}$:

$$M_{a,b}(t) = \sum_{i=0}^{a} \sum_{j=0}^{b} f_{i,j}^{a,b} \cdot p_{\mathsf{A}}(t)^{a-i} \cdot p_{\mathsf{B}}(t)^{b-j} \cdot M_{i,j}(t-1) \tag{4}$$

Now that we are given the equations for computing an entry in the fingerprint at a specific synthesis step for both models without reactivity parameters, we can show that an inversion of neighboring values in p_{A} does not change the resulting fingerprint.

Lemma 1. *Given the Bernoulli model without reactivity parameters and a permutation $\pi(p_A)$ that swaps $p_A(t)$ with $p_A(t-1)$, then $M_{a,b}(t) = M^{\pi}_{a,b}(t)$ holds for all $a > 0$, $b > 0$, and $2 \leq t \leq T$.*

Proof. Inserting $M_{a,b}(t-1)$ into the recursive equation 1 yields:

$$\begin{aligned}
M_{a,b}(t) = \quad & p_M^2 \cdot p_{\mathsf{A}}(t) \cdot p_{\mathsf{A}}(t-1) \cdot M_{a-2,b}(t-2) \\
+ \quad & p_M^2 \cdot p_{\mathsf{B}}(t) \cdot p_{\mathsf{B}}(t-1) \cdot M_{a,b-2}(t-2) \\
+ \quad & p_M^2 \cdot p_{\mathsf{A}}(t) \cdot p_{\mathsf{B}}(t-1) \cdot M_{a-1,b-1}(t-2) \\
+ \quad & p_M^2 \cdot p_{\mathsf{B}}(t) \cdot p_{\mathsf{A}}(t-1) \cdot M_{a-1,b-1}(t-2) \\
+ \quad & p_M \cdot (1-p_M) \cdot p_{\mathsf{A}}(t) \cdot M_{a-1,b}(t-2) \\
+ \quad & p_M \cdot (1-p_M) \cdot p_{\mathsf{A}}(t-1) \cdot M_{a-1,b}(t-2) \\
+ \quad & p_M \cdot (1-p_M) \cdot p_{\mathsf{B}}(t) \cdot M_{a,b-1}(t-2) \\
+ \quad & p_M \cdot (1-p_M) \cdot p_{\mathsf{B}}(t-1) \cdot M_{a,b-1}(t-2) \\
+ \quad & (1-p_M)^2 \cdot M_{a,b}(t-2)
\end{aligned} \tag{5}$$

We replace $p_{\mathsf{A}}(t-1)$ with $\pi(p_{\mathsf{A}})(t)$, $p_{\mathsf{A}}(t)$ with $\pi(p_{\mathsf{A}})(t-1)$, $p_{\mathsf{B}}(t-1)$ with $\pi(p_{\mathsf{B}})(t)$, and $p_{\mathsf{B}}(t)$ with $\pi(p_{\mathsf{B}})(t-1)$:

$$\begin{aligned} M_{a,b}(t) = &\quad p_M^2 \cdot \pi(p_{\mathsf{A}})(t) \cdot \pi(p_{\mathsf{A}})(t-1) \cdot M_{a-2,b}(t-2) \\ + &\quad p_M^2 \cdot \pi(p_{\mathsf{B}})(t) \cdot \pi(p_{\mathsf{B}})(t-1) \cdot M_{a,b-2}(t-2) \\ + &\quad p_M^2 \cdot \pi(p_{\mathsf{A}})(t) \cdot \pi(p_{\mathsf{B}})(t-1) \cdot M_{a-1,b-1}(t-2) \\ + &\quad p_M^2 \cdot \pi(p_{\mathsf{B}})(t) \cdot \pi(p_{\mathsf{A}})(t-1) \cdot M_{a-1,b-1}(t-2) \\ + &\quad p_M \cdot (1-p_M) \cdot \pi(p_{\mathsf{A}})(t) \cdot M_{a-1,b}(t-2) \\ + &\quad p_M \cdot (1-p_M) \cdot \pi(p_{\mathsf{A}})(t-1) \cdot M_{a-1,b}(t-2) \\ + &\quad p_M \cdot (1-p_M) \cdot \pi(p_{\mathsf{B}})(t) \cdot M_{a,b-1}(t-2) \\ + &\quad p_M \cdot (1-p_M) \cdot \pi(p_{\mathsf{B}})(t-1) \cdot M_{a,b-1}(t-2) \\ + &\quad (1-p_M)^2 \cdot M_{a,b}(t-2) \end{aligned} \tag{6}$$

We simplify the equation to:

$$\begin{aligned} M_{a,b}(t) = &\quad p_M \cdot \pi(p_{\mathsf{A}})(t) \cdot M_{a-1,b}(t-1) \\ + &\quad p_M \cdot \pi(p_{\mathsf{B}})(t) \cdot M_{a,b-1}(t-1) \\ + &\quad (1-p_M) \cdot M_{a,b}(t-1) \end{aligned} \tag{7}$$

Which can be further simplified to:

$$M_{a,b}(t) = M^{\pi}_{a,b}(t) \tag{8}$$

□

Lemma 2. *Given the Geometric model without reactivity parameters and a permutation $\pi(p_A)$ that swaps $p_A(t)$ with $p_A(t-1)$, then $M_{a,b}(t) = M^{\pi}_{a,b}(t)$ holds for all $a > 0$, $b > 0$, and $2 \le t \le T$.*

Proof Sketch. Inserting $M_{i,j}(t-1)$ into the recursive equation 4 yields:

$$\begin{aligned} M_{a,b}(t) = &\sum_{i=0}^{a}\sum_{j=0}^{b} f^{a,b}_{i,j} \cdot p_{\mathsf{A}}(t)^{a-i} \cdot p_{\mathsf{B}}(t)^{b-j} \\ &\cdot \sum_{k=0}^{i}\sum_{l=0}^{j} f^{i,j}_{k,l} p_{\mathsf{A}}(t-1)^{i-k} \cdot p_{\mathsf{B}}(t-1)^{j-l} \cdot M_{k,l}(t-2) \end{aligned} \tag{9}$$

Writing the terms of the sums explicitly yields a large equation of the following form:

$$\begin{aligned} M_{a,b}(t) = &f^{a,b}_{0,0} p_{\mathsf{A}}(t)^a p_{\mathsf{B}}(t)^b \\ &+ f^{a,b}_{0,1} p_{\mathsf{A}}(t)^a p_{\mathsf{B}}(t)^{b-1} \left(f^{0,1}_{0,0} M_{0,0}(t-2) + f^{0,1}_{0,1} p_{\mathsf{B}}(t) M_{0,1}(t-2) \right) \\ &+ f^{a,b}_{1,0} p_{\mathsf{A}}(t)^{a-1} p_{\mathsf{B}}(t)^b \left(f^{1,0}_{0,0} M_{0,0}(t-2) + f^{1,0}_{1,0} p_{\mathsf{A}}(t) M_{1,0}(t-2) \right) \\ &+ f^{a,b}_{1,1} p_{\mathsf{A}}(t)^{a-1} p_{\mathsf{B}}(t)^{b-1} \left(f^{1,1}_{0,0} M_{0,0}(t-2) + f^{1,1}_{1,0} p_{\mathsf{A}}(t) M_{1,0}(t-2) \right. \\ &\qquad \left. + f^{1,1}_{0,1} p_{\mathsf{B}}(t) M_{0,1}(t-2) + f^{1,1}_{1,1} p_{\mathsf{A}}(t) p_{\mathsf{B}}(t) M_{1,1}(t-2) \right) \\ &+ \dots \\ &+ f^{a,b}_{a,b} \left(f^{a,b}_{0,0} M_{0,0}(t-2) + \dots + f^{a,b}_{a,b} p_{\mathsf{A}}(t-1)^a p_{\mathsf{B}}(t-1)^b M_{a,b}(t-2) \right) \end{aligned} \tag{10}$$

If we now expand this equation, we see that for every term of the form $p_{\mathsf{A}}(t)^{\alpha} p_{\mathsf{B}}(t)^{\beta} p_{\mathsf{A}}(t-1)^{\gamma} p_{\mathsf{B}}(t-1)^{\delta}$ there is a corresponding term $p_{\mathsf{A}}(t)^{\gamma} p_{\mathsf{B}}(t)^{\delta} p_{\mathsf{A}}(t-1)^{\alpha} p_{\mathsf{B}}(t)^{\beta}$ and we change equation 9 to:

$$M_{a,b}(t) = \sum_{i=0}^{a}\sum_{j=0}^{b} f_{i,j}^{a,b} \cdot p_{\mathsf{A}}(t-1)^{a-i} \cdot p_{\mathsf{B}}(t-1)^{b-j} \cdot \sum_{k=0}^{i}\sum_{l=0}^{j} f_{k,l}^{i,j} p_{\mathsf{A}}(t)^{i-k} \cdot p_{\mathsf{B}}(t)^{j-l} \cdot M_{k,l}(t-2) \tag{11}$$

We replace $p_{\mathsf{A}}(t-1)$ with $\pi(p_{\mathsf{A}})(t)$, $p_{\mathsf{A}}(t)$ with $\pi(p_{\mathsf{A}})(t-1)$, $p_{\mathsf{B}}(t-1)$ with $\pi(p_{\mathsf{B}})(t)$, and $p_{\mathsf{B}}(t)$ with $\pi(p_{\mathsf{B}})(t-1)$:

$$M_{a,b}(t) = \sum_{i=0}^{a}\sum_{j=0}^{b} f_{i,j}^{a,b} \cdot \pi(p_{\mathsf{A}})(t)^{a-i} \cdot \pi(p_{\mathsf{B}})(t)^{b-j} \cdot \sum_{k=0}^{i}\sum_{l=0}^{j} f_{k,l}^{i,j} \pi(p_{\mathsf{A}})(t-1)^{i-k} \cdot \pi(p_{\mathsf{B}})(t-1)^{j-l} \cdot M_{k,l}(t-2) \tag{12}$$

We simplify the equation to:

$$M_{a,b}(t) = M_{a,b}^{\pi}(t) \tag{13}$$

□

For the models without reactivity parameters, we know from Lemma 1 and 2 that no inversion of neighboring values in p_{A} changes the resulting fingerprint. Any permutation of a vector can be constructed by a sequence of inversions of neighboring elements. Therefore, for the Bernoulli and Geometric model without reactivity parameters, all permutations of a probability vector p_{A} have the same resulting fingerprint.

3. Parameter optimization

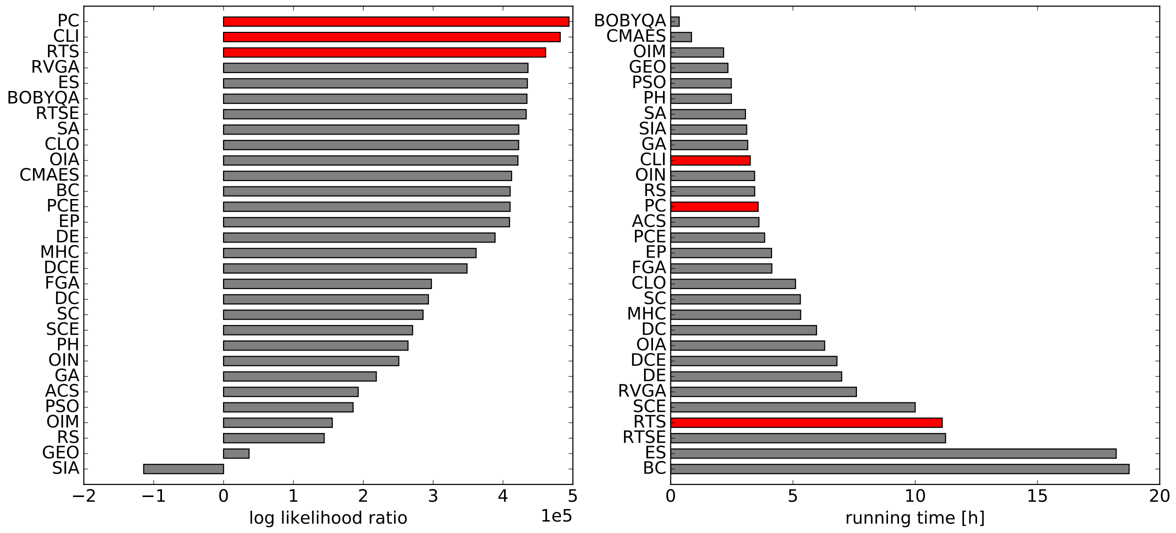

Figure S2. Left: Log likelihood ratio of all optimization algorithms using the direct method on the $DP_n = 3, r_{\mathsf{A}} = 2.0$ instance without noise. The top three algorithms are marked in red. Right: Running times of the algorithms.

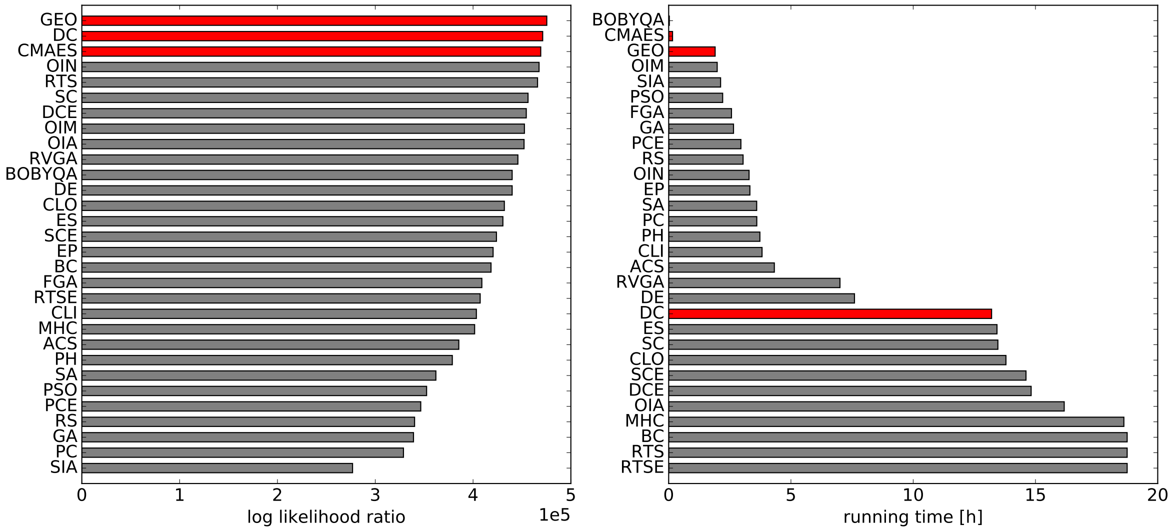

Figure S3. Left: Log likelihood ratio of all optimization algorithms using the spline method on the $DP_n = 3, r_\mathrm{A} = 2.0$ instance without noise. The top three algorithms are marked in red. Right: Running times of the algorithms.

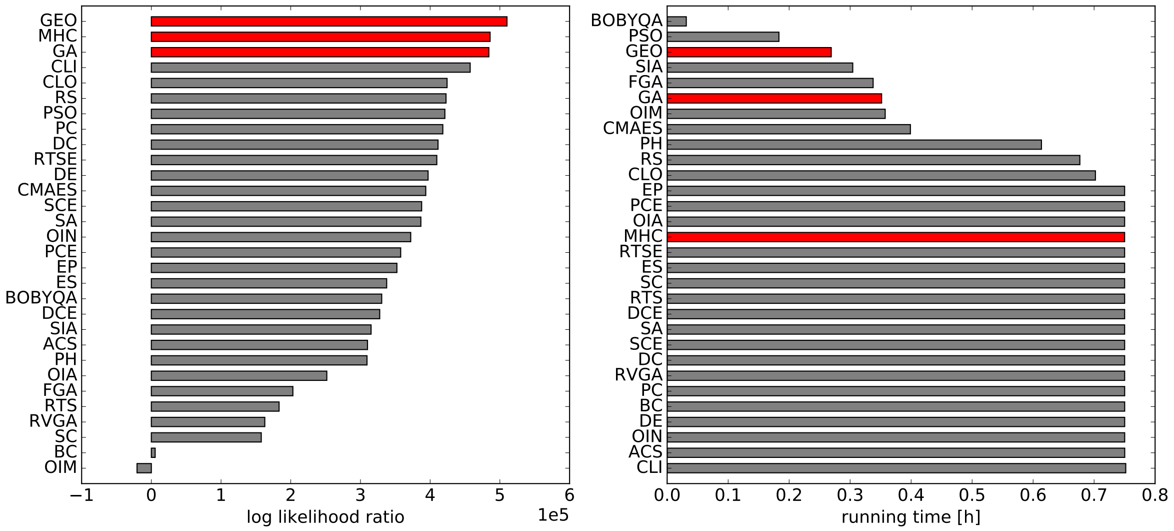

Figure S4. Left: Log likelihood ratio of all optimization algorithms using the ODE method on the $DP_n = 3, r_\mathrm{A} = 2.0$ instance without noise. The top three algorithms are marked in red. Right: Running times of the algorithms.

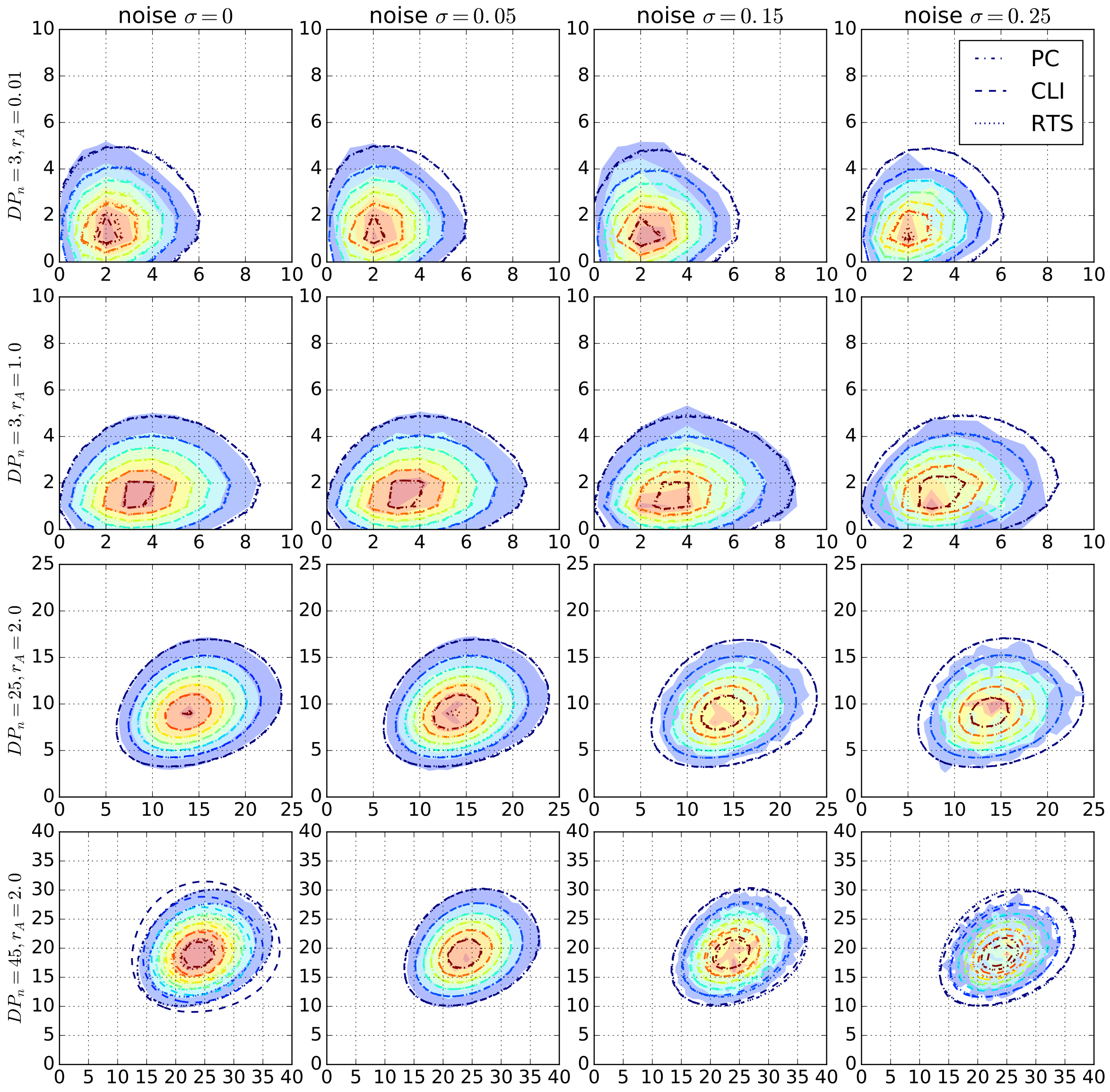

Figure S5. Filled contours: Fingerprints of the datasets $DP_n = 3, r_{\mathrm{A}} = 0.01$, $DP_n = 3, r_{\mathrm{A}} = 1.0$, $DP_n = 25, r_{\mathrm{A}} = 2.0$, and $DP_n = 45, r_{\mathrm{A}} = 2.0$, (top to bottom) with increasing noise (left to right). Contours: Fingerprints computed by the model using the direct method.

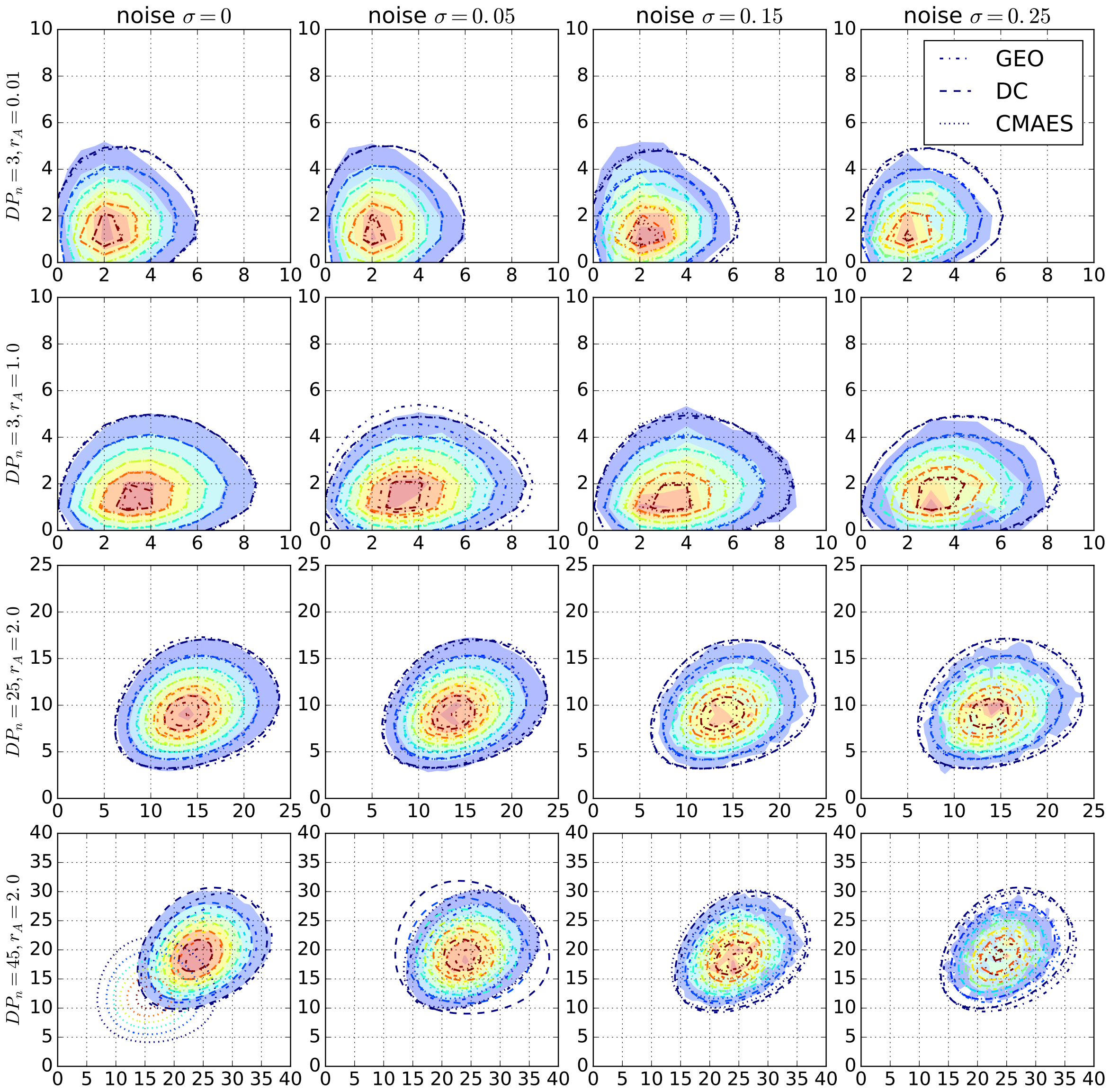

Figure S6. Filled contours: Fingerprints of the datasets $DP_n = 3, r_{\mathrm{A}} = 0.01$, $DP_n = 3, r_{\mathrm{A}} = 1.0$, $DP_n = 25, r_{\mathrm{A}} = 2.0$, and $DP_n = 45, r_{\mathrm{A}} = 2.0$, (top to bottom) with increasing noise (left to right). Contours: Fingerprints computed by the model using the spline method.

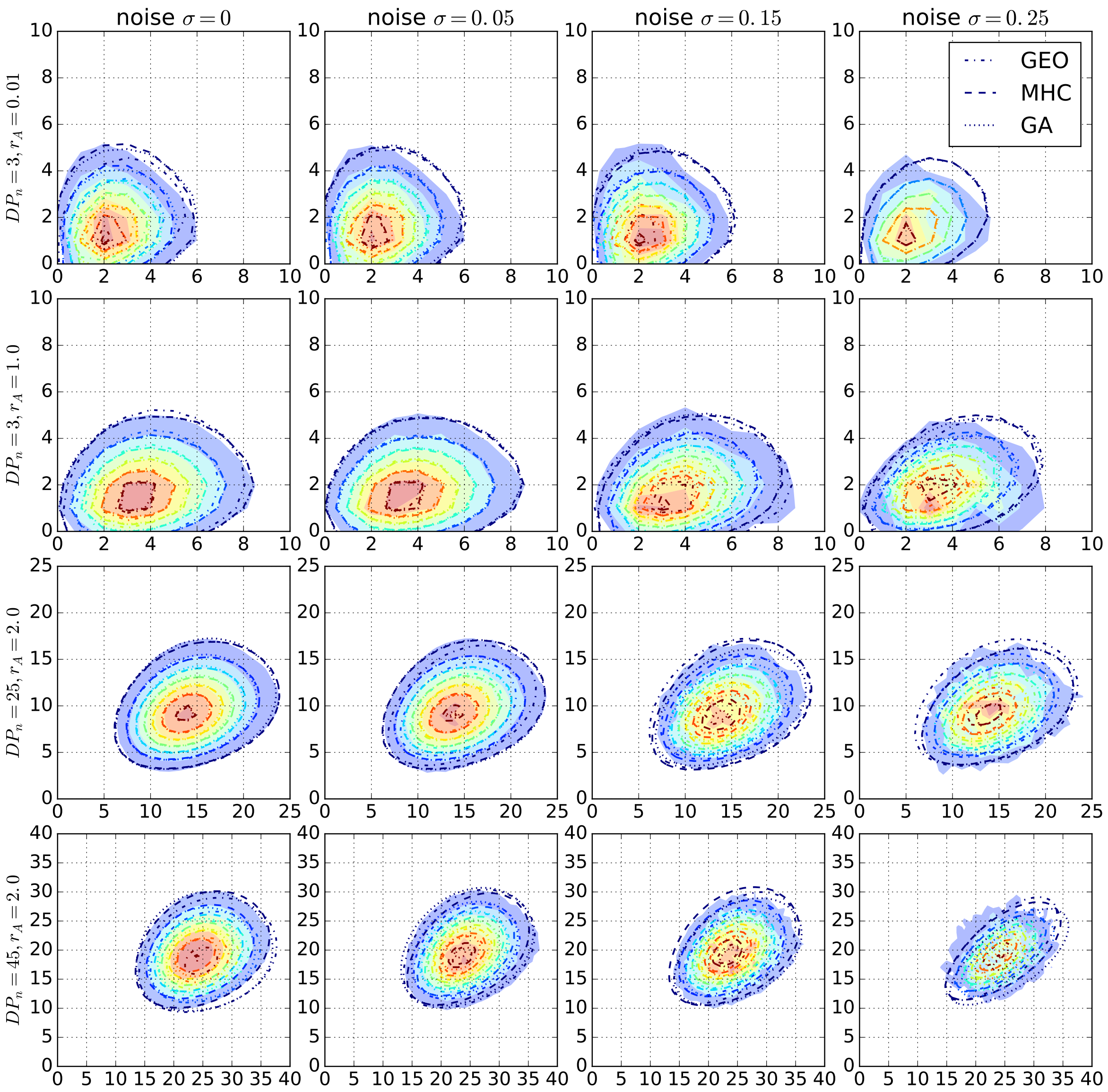

Figure S7. Filled contours: Fingerprints of the datasets $DP_n = 3, r_\mathrm{A} = 0.01$, $DP_n = 3, r_\mathrm{A} = 1.0$, $DP_n = 25, r_\mathrm{A} = 2.0$, and $DP_n = 45, r_\mathrm{A} = 2.0$, (top to bottom) with increasing noise (left to right). Contours: Fingerprints computed by the model using the ODE method.

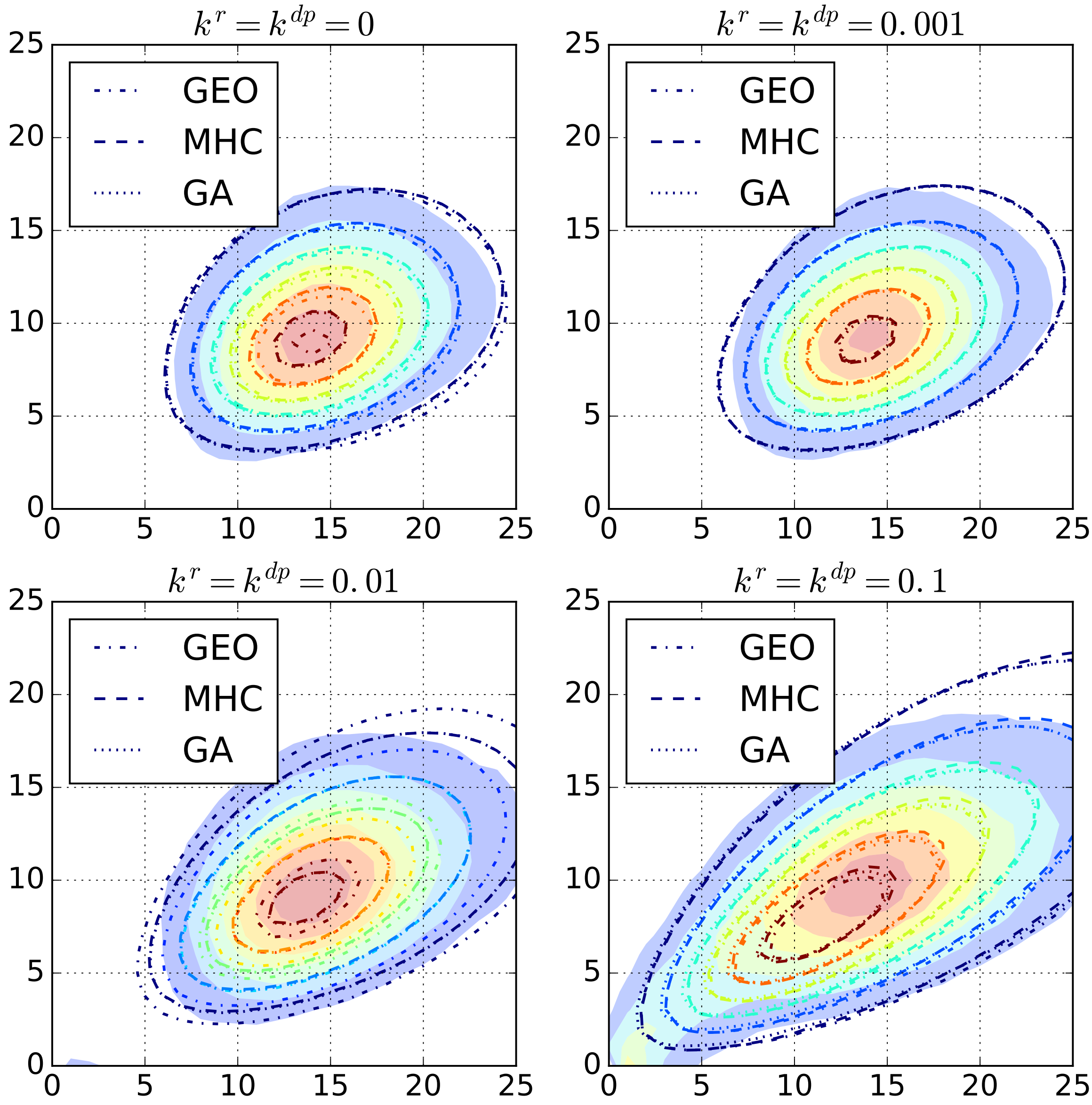

Figure S8. Filled contours: Fingerprints of the controlled radical copolymerizations with increasing recombination k^r and disproportionation rates k^{dp}. Contours: Fingerprints computed by the model using the ODE method.

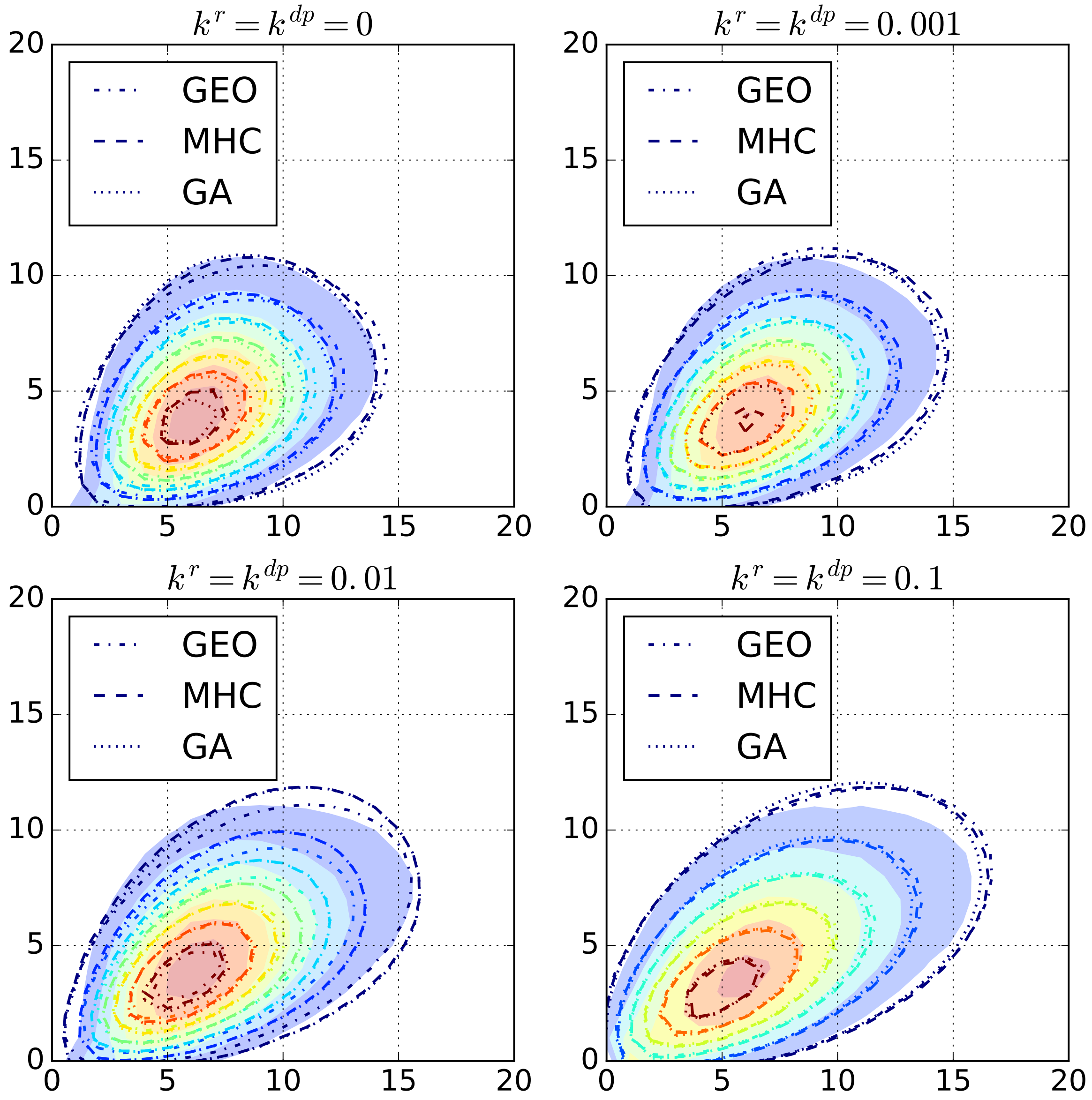

Figure S9. Filled contours: Fingerprints of the free radical copolymerizations with increasing recombination k^r and disproportionation rates k^{dp}. Contours: Fingerprints computed by the model using the ODE method.

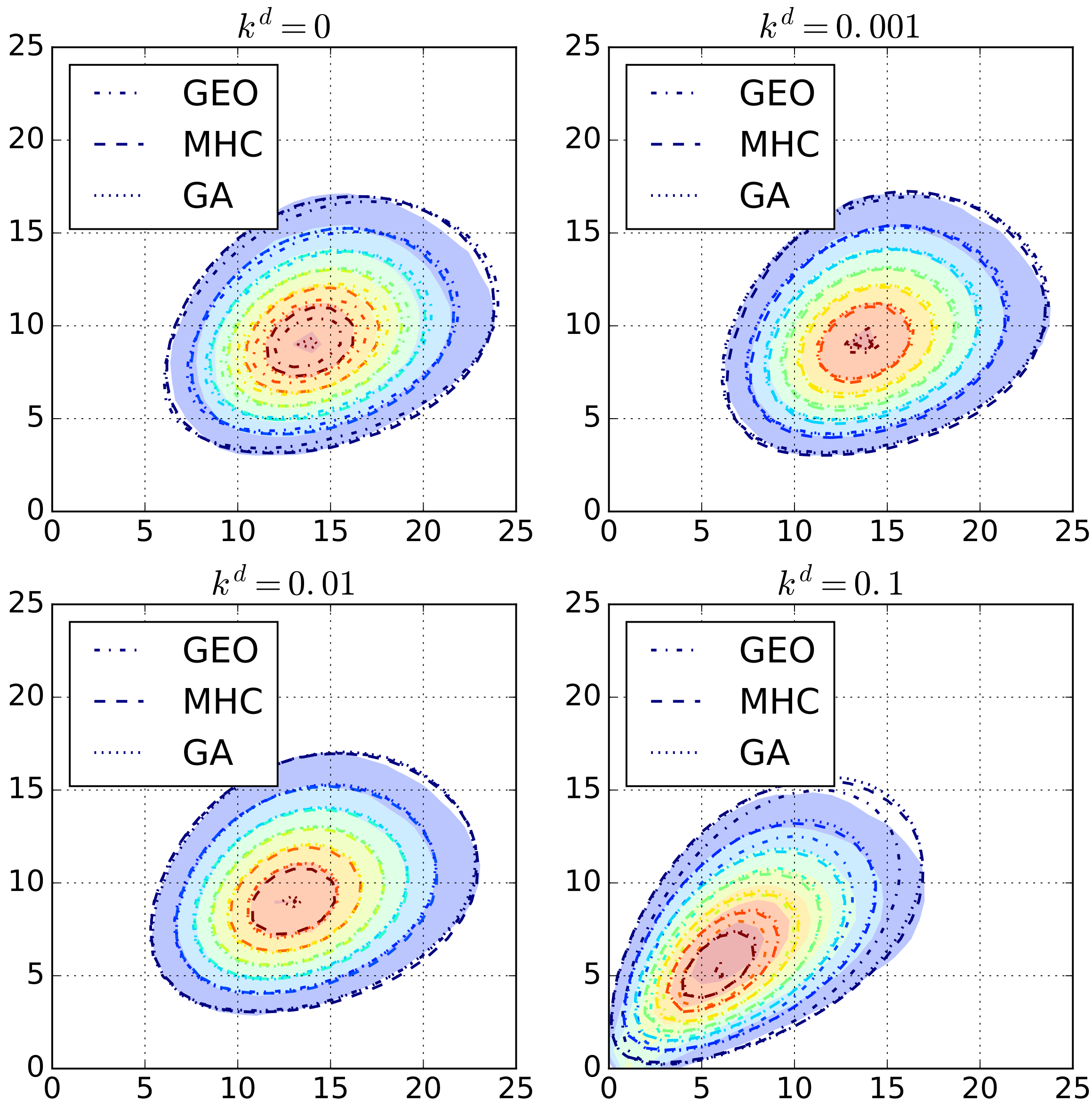

Figure S10. Filled contours: Fingerprints of the reversible living copolymerizations with increasing depropagation rates k^d. Contours: Fingerprints computed by the model using the ODE method.

Bibliography

1. Engler, M.S.; Scheubert, K.; Schubert, U.S.; Böcker, S. New Statistical Models for Copolymerization. *Polymers* **2016**, *8*, 240.
